# Evacuate or Stay? A Typhoon Evacuation Decision Model in China Based on the Evolutionary Game Theory in Complex Networks

**DOI:** 10.3390/ijerph17030682

**Published:** 2020-01-21

**Authors:** Dian Sun, Lupeng Zhang, Zifeng Su

**Affiliations:** 1 School of Public Policy & Management, Tsinghua University, Beijing 100084, China; sundian900621@163.com; 2 Center for Crisis Management Research (Sponsored by Beijing Planning Office of Philosophy & Social Science), Tsinghua University, Beijing 100084, China; 3 School of Public Administration, Beijing University of Aeronautics and Astronautics, Beijing 100191, China; 4 School of Economics, Peking University, Beijing 100871, China; suzifeng@cdb.cn; 5 Finance Research and Development Center, China Development Bank, Beijing 100032, China

**Keywords:** emergency evacuation, evacuation decision-making, evolutionary game theory, evacuees

## Abstract

The Chinese Government has played an important role in organizing the evacuation of typhoon disasters, and in-depth analysis of individual behavioral decisions is a prerequisite for adopting an effective emergency organization plan. Existing evacuation plans only consider how the Government issues the early warning and organizes the mandatory evacuation, but does not formulate effective policies to improve the efficiency of self-evacuation of evacuees and lacks the understanding of individual evacuation decision-making. Using game-based theory in a small-world network context, we build an evolutionary game model of evacuation decision diffusion between evacuees in the context of a complex network. The model simulates the effects of guaranteeing the evacuation order and providing material supplies on the evacuation decision diffusion in a small-world network in China. The results showed that various levels of policy-implementation led to different rates of evacuation. As the cost-reduction of the evacuation process increased, the evacuation response rate in the social system increased. In contrast, as the rate of reducing the non-evacuation cost decreased or the cost-reduction rate of non-evacuation increased, the evacuation response rate in the social system decreased. The study findings provided insights on emergency planning and the effectiveness of their implementation in social networks, which can be used to improve evacuation policy.

## 1. Introduction

Typhoons, which are high-frequency natural disasters, have caused huge losses to countries around the world in recent years and created widespread concern. China is one of the countries that have suffered the most from frequent typhoon disasters. According to the data released by the Central Weather Bureau, from 1949 to 2018, on average, we have to be prepared for the threat of seven typhoons every year. China, which has been severely affected by geographical conditions and climatic conditions, has incurred substantial losses due to typhoon disasters while enjoying abundant rainfall every year. Since 2000, typhoons have killed about 200 people and caused direct economic losses of more than 46 billion yuan every year, accounting for 27% of the total direct economic losses from floods. However, when people take effective protective measures such as evacuation against predictable typhoon disasters, the serious consequences can be greatly reduced. In 2016 alone, China was hit by 29 typhoons, and approximately 700,000 people were evacuated. Under Typhoon Lekima in 2019, more than two million people were resettled.

When a severe typhoon is imminent, people in danger zones usually make evacuation decisions based on the information they have. This process is subjective and integrated with individual characteristics. However, considering evacuation costs, family property, and other unpredictable risks, some evacuees adopt adventurous strategies to stay at home [1], virtually guaranteeing a negative impact on themselves, their families, and even the country. Other residents in safe areas participate in the evacuation in panic because of misunderstanding and a lack of knowledge about disaster prevention, which has in the past interfered with evacuation orders and consumed valuable evacuation resources [2]. As a result, one of the main goals of the Chinese Government’s emergency management policies has been to adopt rescue measures, such as evacuating and properly relocating people who are threatened by predictable natural disasters. Therefore, it is important to explore the decision-making and willingness of evacuated groups under the influence of typhoon disasters in order to improve China’s emergency evacuation plan and promote emergency management development.

Based on the micro-perspective analysis, although the Government’s issue evacuation orders (voluntary or mandatory), the final decision-making power lies with evacuated groups in dangerous areas. Competition among evacuees for evacuation resources often drives their behavioral decisions, resulting in different outcomes [3]. At the same time, the evacuated individuals consider the behavior of other participants in decision-making [4]. Other people’s decisions affect the perceived payoff of their own decisions, and they often imitate the decisions of their close contacts. Therefore, policy makers cannot expect all people to fully adhere to evacuation orders [5]. At present, there are some studies have employed game strategy to understand real behavioral interactions between individual evacuees [6]. They often mode and simulate crowd behaviors by employing agent-based model [6,7] and consider an evacuation process based on a game-theoretical approach [8,9], but the individual interactions through social networks are often ignored in their models. In addition, there are relatively fewer studies on the influences of, and changes, in evacuee decision-making under the condition of government participation during the large-scale evacuation process. Therefore, this study utilized game theory to examine the possible power struggles and trade-offs of interests between multiple stakeholders. In addition, the study adopted the complex-network theory to deal with the structural characteristics of evacuees who communicated social networks so that to explain the mechanism of evacuation decision diffusion in the social network. Based on these theories, this study constructed an evolutionary game model in complex network. We analyzed the decision-making mechanism of evacuations under typhoons when the Government was involved focusing on two questions: Q1: What is the mechanism of interaction between decision-making individuals in response to typhoon disasters? Q2: How do policies impact the decision-making of emergency evacuation? Based on these questions, we proposed policy implications for Chinese evacuation planning makers and academics.

The remainder of the paper is organized as follows. Section 2 provides a review of the related literature, based on which we develop the analytical framework. Section 3 discusses the evolutionary game model in the complex network that has been established to explain the mechanism of evacuation diffusion in the social network. This is followed by a numerical simulation and discussion to analyze how Government involvement impact the diffusion in Section 4. In Section 5, conclusions and suggestions are provided.

## 2. Literature Review

### 2.1. Evacuation Decision-Making

Understanding the evacuation decision of a potential evacuee is the key to the success of an emergency evacuation plan. The evacuation decision is mainly based on the type of order (voluntary evacuation or mandatory evacuation with Government participation), trust in evacuation instructions, perception of risk, and subjective approval of the evacuation plan [10,11]. It is also subject to the influence of social factors and resource availability. Until now, research on evacuation decision-making has mainly focused on the evacuation warnings and information dissemination, zoning, demand modeling, traffic assignment and route selection, shelter-seeking, evacuation strategies and logistical issues [11,12,13,14,15,16,17,18]. Research on whether to participate in evacuation has mostly been conducted from the individual point of view. Through the use of survey data, logistic regression models have been constructed to determine the factors that influence decision-making [19,20], or artificial neural network models have been constructed from a group perspective to predict traffic demand [21].

Most research on typhoon evacuation decision-making has been based on identifying different typhoon situations, surveying urban residents, and analyzing and discussing the influencing factors of behaviors under the influence of typhoon disasters [22,23,24,25]. Studies have shown that proximity to the typhoon, the likelihood of the threat occurrence, warning source and risk perception significantly influence the people’s decision-making [4,16,17]. In different situations, the evacuation behaviors of evacuated people in the same area were inconsistent; similarly, in the same situation, the evacuated groups in different regions also had exhibited evacuation behaviors, and even in the same regions, the different processing and interpretation of information by individuals will lead to different compliance of evacuation instructions [26]. The evacuation behaviors were divided into two categories: a group that only considered the individual behavior, and a group that considered the group activity behavior as the research object. The constructed model also contained more subtle variables related to behavior. These studies on crowd evacuation behavior evidenced that most research was based on the assumption that random utility is maximized and used the statistical model of the questionnaire data to identify the factors affecting people’s evacuation decision-making and find out the steps of behavioral patterns of the evacuated population. However, there have been few studies delving into the interactions between these individuals. In addition, China’s national conditions are such that large-scale evacuation is mainly organized by the Government. The academic research on emergency evacuation has mostly focused on the micro-individual evacuation level of large buildings and distribution centers [27,28]. Thus, our study analyzed decision-making behavior in the case of large-scale regional evacuation of individuals with Government participation.

### 2.2. Social Cues of Evacuation

According to the research of Lindell et al., the impact of the evacuation regional environment includes both the natural environment and the social environment. This includes the environmental cues and social cues in the PADM (Protective Action Decision Model) model and the individual’s subjective interpretation of the environment, which leads to protective behavior [29]. The natural environment referred to the danger of geophysical, meteorological, hydrological, or technological processes arising from typhoon disasters, for instance, environmental cues such as typhoon intensity and the evacuee’s distance from the typhoon and from the evacuation destination. These factors directly affect the public perception of risk, which affects evacuee decision-making behavior. According to Fu et al. research, environmental factors such as typhoon distance and wind speed are used as variables in the evacuation decision-making selection model [30].

In the social environment, factors that promote and prevent people in adopting protective behaviors are classified into social environmental factors, such as the influence of other people’s behaviors on the behavioral decisions of the evacuees. These include the evacuation behavior of friends or relatives, which strongly promotes the evacuation decision of the evacuees. This is because under the condition of risk uncertainty, it is common to observe the actions of others before making decisions, as is the case with conformity behavior. Studies have indicated that neighbors’ evacuation decisions positively correlated with individual evacuation decisions [31], whereas other studies suggested that individual evacuation decisions are influenced by other people’s decisions through pro-social networks [32]. In addition, social environmental factors also include factors that hinder people from taking protective actions, including the impact of other people’s decision-making on the cost of individual evacuation or non-evacuation costs. These factors include expenses and time cost, which are social environmental factors that hinder evacuation. The models for evacuation behavior prediction are based on the set model, which uses the logistic regression [20], neural network [33], and ordered logit models. The models can be validated by estimating the model and collecting the evacuation behavior selection data to predict the evacuation traffic demand. However, these models ignore the independence and mutual influence between individuals. A relatively new method to solve this kind of distribution-control problem is represented by Han Qi et al., who combined game theory and discrete selection theory with stochastic optimal reaction equilibrium theory to study the time-decision mechanism of individual daily travel and proposed the scope of application used in our method [34].

### 2.3. Role of Government

When the Government issues early warnings, such as evacuation orders and traffic information, information including route selection and evacuation schedules can make evacuation operations more orderly, but policy makers cannot expect all people to fully obey orders [5]. Studies have indicated that when the Government organizes public transportation system to help evacuate people, prepare for the evacuation activities, allocate emergency materials needed for disasters [35], and formulate policies for post-disaster reconstruction, it can help the evacuated groups reduce their evacuation costs or costs associated with staying to some extent during the disaster, which allows evacuation operations to be carried out efficiently and to maximize the protection of life and property. Borowska-Stefańskaa et al. emphasized the important role of evacuation, and develop an optimization pattern for the evacuation process before the flood, which could be used for the development of a policy related to the management of flood risk in order to increase the efficiency of the evacuation [35]. Batty et al. studied how the evacuated small-scale vehicles develop into large-scale group evacuation behaviors and carried out simulation research to explore the positive role of traffic-control measures in the emergency evacuation process [36]. Similarly, Swamy et al. focused on the problem of public transportation routes in the process of evacuation and constructed an optimization model for using public transportation to carry out emergency evacuation of typhoons, which provided a reference for the Government to formulate plans [37]. In addition, Gong established an evolutionary game model of travel-route selection behavior under information-induced conditions to support the establishment of traffic-inducing information strategies [38].

Previous research was mostly adopted from a micro or macro perspective to study the decision-making effect of different influencing factors on evacuated individuals and changes in evacuation demand, as well as influence of the channels and methods of government information dissemination on the evacuation decision-making of the evacuated individuals. However, the development of social media has increased the frequency of interaction between individuals after disasters occur, and the effect of mutual influences between individual decisions has been prominent; thus, the social network environment is an external environment that cannot be ignored in the process of decision-making of evacuated individuals. In China, the Government’s response to disasters involves more than just information release. The Government formulates emergency evacuation plans and takes measures to protect individual lives and property to the greatest extent possible. In contrast with existing research, we used evolutionary game theory to analyze how Government involvement affects the behavior of evacuees in terms of the diffusion of evacuation decision-making, and we expounded on this issue in combination with complex network theory to generalize the conclusions.

## 3. Research Method

The purpose of the network evolutionary game is to simulate the process of evacuation decision-making in complex network. Therefore, we have two steps to construct the model. Firstly, we must ensure the evolutionary game which is represent the evacuee’s behavior, that is evacuate to the safety zone or stay at home, also include the profit (cost saving) of each evacuee under different policies and their behaviors. Secondly, we had better choose the environment that the dynamic evolution happened, particularly we should design a complex network which shows the true structure of evacuees’ information exchanging during typhon disaster.

### 3.1. Model of Evolutionary Game

#### 3.1.1. Basic Assumptions of the Evolutionary Game Model

Before constructing the model, we considered the assumptions of the model that might limit our network evolutionary game.

**Assumption 1:** In an area that is likely to be affected by typhoon disasters, the evacuated groups need to compete for certain social resources (e.g., highway road resources, oil and gas resources, and emergency facilities resources) to maximize their own interests. There are two types of players: individuals in the social network, and the Government. All the evacuees are under the threat of a typhoon and receive an evacuation warning.

**Assumption 2:** After receiving evacuation warning, each player has two possible courses of action. They can choose to evacuate to the safety zone or stay at home, so the strategy set is {Evacuate, Stay}. The evacuees’ aim is to make the decision that maximizes its own interests. However, the evacuees are assumed to have limited rationality and incomplete information. Therefore, they cannot always make the choice that maximizes their own interests.

**Assumption 3:** The Government has three kinds of policies to choose from: fixed asset loss allowance, improving emergency evacuation plan to save evacuation cost, and providing material support to save on the cost of non-evacuation. The Government usually uses different evacuation planning tools to improve compliance of evacuation command and ensure the individuals who stay at home are safety.

**Assumption 4:** Based on the analysis above, evacuation decision-making diffusion occurs in a small world complex network which represents the social network of evacuees’ information exchanging. A payoff matrix is established based on evolutionary game theory (Friedman, 1998) as shown in Table 1.

#### 3.1.2. Notation

The earnings matrix among two evacuees is established in Table 1. In Table 1, $U_{ij}$ and $V_{ij}$ represent the profits of the evacuees which make a decision of different strategies (evacuate or stay), respectively, where:

(1) $$U_{11}=P_{1}-\alpha (C_{1}-{C_{1}}^{\prime })-(1-\delta )D_{1}$$

(2) $$V_{11}=P_{2}-\alpha (C_{2}-{C_{2}}^{\prime })-(1-\delta )D_{2}$$

(3) $$U_{12}=P_{1}-\alpha (C_{1}-{C_{1}}^{\prime })-(1-\delta )(1-\beta )D_{1}$$

(4) $$V_{12}=(1-\alpha )P_{2}-\varepsilon (1-\theta )E_{2}$$

(5) $$U_{21}=(1-\alpha )P_{1}-\varepsilon (1-\theta )E_{1}$$

(6) $$V_{21}=P_{1}-\alpha (C_{1}-{C_{1}}^{\prime })-(1-\delta )(1-\beta )D_{1}$$

(7) $$U_{22}=(1-\alpha )P_{1}-\eta E_{1}$$

(8) $$V_{22}=(1-\alpha )P_{2}-\eta E_{2}$$

In an evacuation process, individuals make evacuation decisions based on their utilities. With limited social resources, individuals need to compete for resources to ensure the maximization of their own interests. The utility value can be determined according to the difference of benefits and costs of each strategy. Considering a typhoon is an emergency situation, we couldn’t get benefit from the disaster, thus the factors which are able to influence the cost of competition for social resources should be considered such as the congestion on the evacuation road, and the availability of emergency supplies. Total assets represent the assessment of individuals’ total benefit if they are not suffering from a typhoon disaster, which used to calculate the individual’s total utility obtained from different strategies.

(1) $P_{i}(i=1,2)$ represents the total assets owned by evacuees, including material property and life property; $C_{i}(i=1,2)$ indicates that in order to ensure the safety of life property when choosing an evacuation strategy, an evacuee abandons a portion of his or her fixed assets.

(2) $D_{i}(i=1,2)$ represents the cost of the evacuation process, including the congestion-time cost, road transportation fee, and accommodation expenses; $E_{i}(i=1,2)$ represents the cost of staying during the typhoon, including the cost of emergency supplies for typhoon disasters, consumed time, housing reinforcement costs, and material procurement expenses.

(3) α represents an individual’s estimate of the probability of a typhoon disaster; $P_{i}-\alpha C_{i}-D_{i}$ is the payoff of both parties choosing the strategy to evacuate without government intervention; and $(1-\alpha )P_{i}-E_{i}$ is the payoff of not choosing the strategy to evacuate. When a person adopts the strategy of not evacuating, social resources are freed up for the evacuated group, road congestion is reduced during the evacuation process, and the cost of evacuation decreases. In addition, β represents the reduced evacuation cost rate, and the cost of the evacuation process is $(1-\beta )D_{i}$. Likewise, the evacuated group gives up social resources to the non-evacuated group and reduces the cost of non-evacuation. Furthermore, θ represents the rate of reducing non-evacuation costs. The cost of not evacuating is $(1-\theta )E_{i}$, where for α, β, θ∈[0,1] the greater α is, the greater the probability that the typhoon disaster may come; the greater β is, the greater the amount of resources that are given up by the non-evacuation group; and the greater θ is, the greater the amount of social resources that are given up by the evacuation group to the non-evacuation group, and vice versa;

(4) When the Government organizes the public transportation system and other means to assist in evacuation (including guidance on the preparation of evacuation activities, the deployment of emergency materials, and the policy of post-disaster reconstruction), evacuated groups can maximize life and property protection to a certain extent. ${C_{i}}^{\prime }$ denotes the reduced loss of fixed assets due to government action during the evacuation process. In addition, δ represents the coefficient of the cost reduction in the evacuation process due to the Government’s organization of public transportation systems to aid in evacuation and the adoption of appropriate strategies to channel traffic; thus, the cost of the evacuation process is $(1-\delta )D_{i}$. In addition, the Government’s measures in the allocation of emergency materials to ensure the supply of materials often lead to lower costs for the original non-evacuation group. However, the Government’s ability is limited, and the protection of the interests of the evacuated groups constrains the Government’s ability to guarantee the interests of the non-evacuation of the group; thus, ε represents the coefficient of the Government’s reduction of the cost of the non-evacuation group compared to the original cost (in terms of commodity value) under the conditions that both groups are guaranteed (one party evacuates but the other does not) and. In addition, η represents the coefficient of the Government’s maximum effort to reduce the cost of the non-evacuation group (both parties do not evacuate) compared to the original cost when the probability of typhoon disasters is low.

According to the aforementioned four strategies, after an information exchange, an evacuated subject with limited rationality would re-evaluate the payoff and adopt the decision-making behavior of whether to evacuate, which means the subjects ate supposed to learn how to play through experience [39]. After a single game, the subject would not withdraw from the social group. Therefore, the heterogeneous subjects would, in the process of the game, continue to learn, try, and make mistakes instead of making one-time choice [40], find the optimal strategy under the influence of social network structure, and then form the inherent evolutionary pattern of the diffusion of decision-making. We used the small-world network structure in a complex network to metaphorize a social network in which relatives and friends exchange information in a social environment [41,42].

### 3.2. Equilibrium Analysis of the Evolutionary Game

There were two groups in the evacuation zone. In the initial stage of the two-player game, we supposed that the proportion of some evacuees choosing to evacuate was x, and the proportion choosing to stay at home was 1−x. Simultaneously, we supposed that the proportion of other evacuees who selected the evacuation strategy was y, and the proportion opting to stay was 1−y, where x∈(0,1) and y∈(0,1). The values of x and y varied with time t.

Suppose that $U_{E1}$ represents the expected earnings of evacuees—Group 1 that choose to evacuate to the shelters, $U_{S1}$ represents the expected earnings of the same group that choose to stay at home, and ${\overset{-}{U}}_{1}$ represents the expected earnings of evacuees—G1 that utilize the both strategies, where,

(9) $$U_{E1}=yU_{11}+(1-y)U_{12}$$

(10) $$U_{S1}=yU_{21}+(1-y)U_{22}$$

(11) $${\overset{-}{U}}_{1}=xU_{E1}+(1-x)U_{S1}$$

Suppose that $U_{E2}$ and $U_{S2}$ are the expected earnings of evacuees—Group 2 that chosen the strategies of evacuate and stay, respectively. ${\overset{-}{U}}_{2}$ indicates the expected earnings of evacuees—G2 that adopted the two strategies, where,

(12) $$U_{E2}=xV_{11}+(1-x)V_{12}$$

(13) $$U_{S2}=xV_{21}+(1-x)V_{22}$$

(14) $${\overset{-}{U}}_{2}=yU_{E2}+(1-y)U_{S2}$$

The replicator dynamic equation of the evacuees—G1 is,

(15) $$\begin{array}{l} f(x,y)=dx/dt=x(1-x)(U_{E1}-U_{S1})\\ \;\;\;=x(1-x)\{y[(\delta -1)\beta D_{1}+E_{1}(1-\theta )\varepsilon -\eta ]\\ \;\;\;\;\;\;\;+[\alpha P_{1}-\alpha (C_{1}-C_{1}^{\prime })-(1-\delta )(1-\beta )D_{1}+E_{1}\eta ]\} \end{array}$$

The replicator dynamic equation of the marginal evacuees—G2 is,

(16) $$\begin{array}{l} g(x,y)=dx/dt=y(1-y)(U_{E2}-U_{S2})\\ \;\;\;=y(1-y)\{x[(\delta -1)\beta D_{2}+E_{2}(1-\theta )\varepsilon -\eta ]\\ \;\;\;\;\;\;\;+[\alpha P_{2}-\alpha (C_{2}-C_{2}^{\prime })-(1-\delta )(1-\beta )D_{2}+E_{2}\eta ]\} \end{array}$$

In the above replicator dynamic system, the equilibrium points (x,y)∈{(x,y)|0≤x≤1,0≤y≤1} include (0,0) (0,1) (1,0) (1,1) $\left(x^{*},y^{*}\right)$.

The Jacobian matrix, J, of the replicator dynamic system is,

(17) $$J=\left[\begin{array}{cc} \frac{\partial f}{\partial x} & \frac{\partial f}{\partial y}\\ \frac{\partial g}{\partial x} & \frac{\partial g}{\partial y} \end{array}\right]\;=\left[\begin{array}{cc} \left(1-2x)[yA_{1}+A_{2}\right] & x(1-x)A_{2}\\ y(1-y)B_{2} & \left(1-2y)(xB_{1}+B_{2}\right) \end{array}\right]$$

where,

(18) $$A_{1}=(\delta -1)\beta D_{1}+E_{1}(1-\theta )\varepsilon -\eta$$

(19) $$A_{2}=\alpha P_{1}-\alpha (C_{1}-C_{1}^{\prime })-(1-\delta )(1-\beta )D_{1}+E_{1}\eta$$

(20) $$B_{1}=(\delta -1)\beta D_{2}+E_{2}(1-\theta )\varepsilon -\eta$$

(21) $$B_{2}=\alpha P_{2}-\alpha (C_{2}-C_{2}^{\prime })-(1-\delta )(1-\beta )D_{2}+E_{2}\eta$$

Namely,

(22) $$\begin{array}{l} DetJ=(1-2x)(1-2y)(yA_{1}+A_{2})(xB_{1}+B_{2})\\ \;\;\;-xy(1-x)(1-y)A_{2}B_{2} \end{array}$$

(23) $$TrJ=(1-2x)(yA_{1}+A_{2})+(1-2y)(xB_{1}+B_{2})$$

According to the determinant (Det) and the trace (Tr) of J, six situations that has five equilibrium points are given in Table 2.

### 3.3. Complex Network Model Construction

We constructed a social network representing the social environment in which the public was located, where G(V,E) is the collection of all nodes in the social network (representing the evacuated individuals), and E is the collection of all edges in the social network. If there was a connecting edge between the two nodes, there was a connection relationship between individuals, so there was a certain probability of generating the behavior of exchanging risk information and thereby affecting the decision-making of both parties.

The small-world network is a common complex network proposed by Watts and Strogatz. The construction principle is as follows: given a coupled network with N nodes, each node is connected to K/2 nodes in its neighboring nodes, each of the original edges in the network is randomly reconnected with probability, P and there must be no redundant edges and self-loops to form a small-world network. The node degree of the small-world network exhibits a Poisson distribution characteristic, and the network has a high clustering coefficient and a short average path length [43]. The evacuated individuals were in a certain topological structure in the social network. After the initial game, the individual, i would randomly select the related neighboring subject, j, to compare the payoffs. Through the game payment matrix shown in Table 1, if the payoff to the individual, i was $pr_{i}<pr_{j}$, would adjust his or her own game strategy and select the strategy of node j according to probability W in the next game [44].

(24) $$W_{i\to j}=\frac{1}{1+\exp[(pr_{j}-pr_{i})/k]}$$

(25) $$p_{ij}=\frac{A_{ij}(t)}{\underset{k\in \Omega_{i}}{\sum A_{ik}(t)}}$$

In Equation (24), k is the noise intensity, indicating the unavoidable interference caused by uncontrollable factors to the subject’s imitation or learning. The larger the k value, the more uncontrollable factors there would be for the individual. We chose the neutral noise intensity, k=1 to perform the simulation. After the individual imitated a decision, the individual selected the information exchange object with a certain probability, $p_{ij}$ to carry out the further diffusion of evacuation behavior. All evacuated individuals in the social network communicated, learned, and chose objects to diffuse according to the above rules. As the number of iterations increased, the individual’s behavior deciding whether to evacuate would reach a stable value. We selected the proportion of the number of subjects in the network that ultimately chose the evacuation behavior in the network to represent the evacuation response rate. This paper describes the different measures used by the Government and the diffusion phenomenon of evacuation decisions under the social network structure with trends in the evacuation response rate.

## 4. The Simulation and Discussion

### 4.1. Parameter Initialization Settings

According to the game analysis of complex network evolution, MATLAB R2016b software (The MathWorks, Inc., Natick, MA, USA) was used to simulate the typhoon disaster evacuation decision-making diffusion. The specific steps were as follows:

Step 1: Generate a small-world network, G(V,E) with a certain number of nodes, and initialize the relevant parameters in the payoff matrix.

Step 2: After the evacuation warning is issued, the evacuate and stay strategies are randomly assigned to each node in the network, represented by 0 or 1.

Step 3: After each round of game, the nodes in the network select neighboring nodes for payoff comparison using the probability shown in Equation (24). If the payoff is greater than or equal to that of the neighbor, the next round game does not make any strategy change; if the payoff is less than the neighbor’s payoff, then the strategy is simulated using the probability, p in Equation (25), and the neighbor’s strategy is selected after the next round of the game;

Step 4: In order to eliminate the interference of randomness on the result, after each simulation is performed 200 times, take the average value and repeat Step 2–Step 4 until the set period *T* is reached. The simulation ends here.

Step 5: Calculate the proportion of subjects that choose the evacuation strategy among all subjects in the network.

Because the nodes in the network represented individuals who were evacuated in society, they were sensitive to government evacuation and security measures and subject to the influence of other individual decisions in the network. Therefore, in order to ensure consistency, we fixed the remaining parameter simulation values and explored the impact of the efficiency of the Government evacuation plan on individual evacuation decisions under the small-world network structure of the metaphorical social network of relatives. Pre-selection of data was done for the evolutionary game analysis before simulation. Initially, the number of subjects in the social network who chose the evacuation and stay strategies was somewhat random. Therefore, using MATLAB software, the initial subject’s strategy selection ratio was randomly assigned. The simulation-related parameters were determined according to the preliminary questionnaire survey and expert interviews. Table 3 shows the scoring by the team at the Center for Emergency Management Research of Tsinghua University, the relevant academics, and experts.

**Figure 1:**
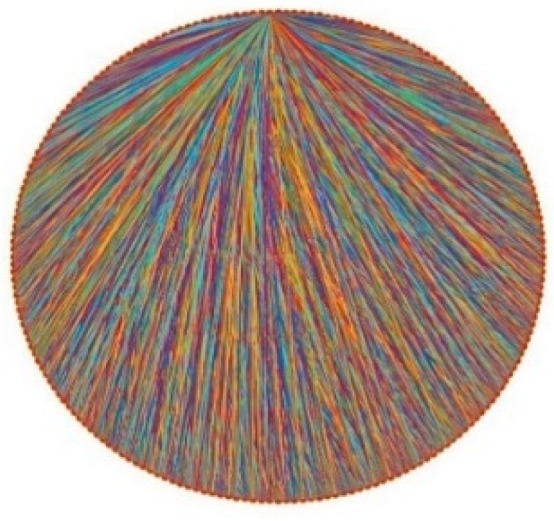
Network of relatives with small-world characteristics.

An initial small-world network with 200 nodes was generated using MATLAB software, is shown in Figure 1.

### 4.2. Numerical Simulation and Discussion

#### 4.2.1. Impact of Decrease in Evacuees on Cost-Reduction Rate Differences in the Evacuation Process

**Figure 2:**
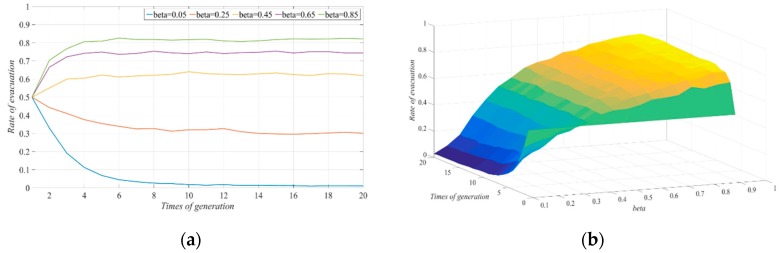
Effect of changes in β on the evolution of evacuation systems: (**a**) two-dimension; (**b**) three-dimension.

The other parameters were fixed in order to explore the impact of the difference in the cost-reduction rate, β caused by the reduction in evacuees during the evacuation process on the selection of evacuation strategy under the typhoon warning conditions. We let the values of β be 0.05, 0.25, 0.45, 0.65, and 0.85 and observed the impact of changes on the evolution of the system. As Figure 2 shows, when the decrease in the number of evacuees resulted in a continuous increase in the cost-reduction rate of the evacuation process, the evacuation response rate in the social system gradually increased, which is similar to the conclusion of previous study [3].

When β was 0.05, the number of people in the evacuation group who chose to evacuate decreased, and these people gradually switched to choosing the non-evacuation strategy. The system finally showed a state of no evacuation; when the value of β was in the range of 0.45–0.85, the evacuation group eventually had a higher proportion of people who chose the evacuation strategy through continuous exchanges and learning. When the value of β was less than 0.45, the decision of the evacuated group was more sensitive to the reduction in the evacuation cost, and when the value of β was greater than 0.45, the number of people who chose to evacuate increased, but they were less sensitive to cost reduction. This was because, in the case of limited social resources, when the social resources given up by the evacuated group who chose not to evacuate decreased, the cost reduction for the individuals who chose the evacuation strategy also decreased. Moreover, after continuous information exchange, the groups in the social system tended to choose not to evacuate. On the contrary, if the non-evacuation strategy could make more social resources available and could greatly reduce the evacuation cost of the evacuated group, then the evacuated individuals in the social system would adopt the evacuation decision. This was reflected in the increase in the evacuation response rate.

#### 4.2.2. Impact on Cost Reduction in Evacuation Process Due to Government Involvement

**Figure 3:**
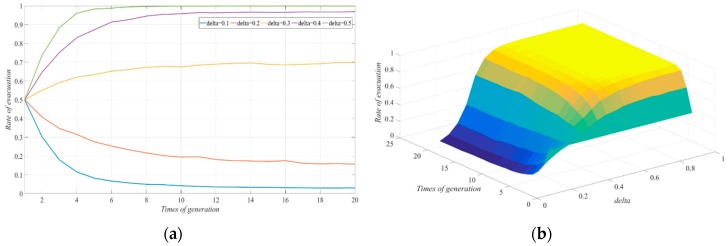
The effect of changes in δ on the evolution of evacuation systems: (**a**) two-dimension; (**b**) three-dimension.

The other parameters were fixed in order to investigate the impact of difference in the cost-reduction rate, δ, in the evacuation process due to the Government’s participation in the typhoon warning conditions. We let δ be 0.1, 0.2, 0.3, 0.4, and 0.5 respectively. The evolutionary results of the strategy selected by the evacuated group in the social network are shown in Figure 3. With an increase in the cost-reduction rate in the evacuation process caused by government participation, the evacuation response rate in the social system gradually increased. When δ was 0.1–0.2, with the continuous exchange of information, the number of people in the evacuated group who chose not to evacuate increased, and the proportion of evacuated people was relatively small. In the end, the social system exhibited a low rate of evacuation response.

When δ was 0.3–0.5, the number of people in the evacuated group who chose the strategy to evacuate gradually increased, and the social system exhibited a high rate of evacuation response. When δ increased to 0.5, the state of all individuals evacuating could be realized. When comparing Figure 2; Figure 3, note that for the same decrease in the cost reduction rate in the evacuation process, when the cost reduction rate under government participation was only between 0.4 and 0.5, there was a stronger impact on the evacuation strategy because of the decrease in cost reduction due to government participation. The corresponding proportion of evacuation was also higher. Therefore, when the effect of cost reduction caused by the Government’s participation in the evacuation process was not obvious, its effect in promoting evacuation decision-making was also not obvious. When the Government took effective measures to participate in the diversion of traffic, such as helping the evacuees choose the evacuation route and arrange evacuation time, the cost of the evacuation process decreased, and its effect was more obvious than the simple reduction of the number of evacuees. Thus, the system would eventually evolve into a full evacuation state. These findings indicated that the Government’s information release mechanism before the arrival of a typhoon can play an important role in the evacuation process. Previous studies have shown that sending warnings judiciously (scientific information release mechanism) could help evacuation efficiency and reduce the cost of congestion in evacuation [45,46]. Thus, effective communication to people in hazardous areas in the process of evacuation can guide and promote the evacuation strategy of the evacuated groups. Effective communication can guide and promote the evacuation strategy of the evacuated groups.

#### 4.2.3. The Degree Coefficient of Government Participation Reduced the Consumption Cost of the Non-Evacuation Group to the Original Cost

**Figure 4:**
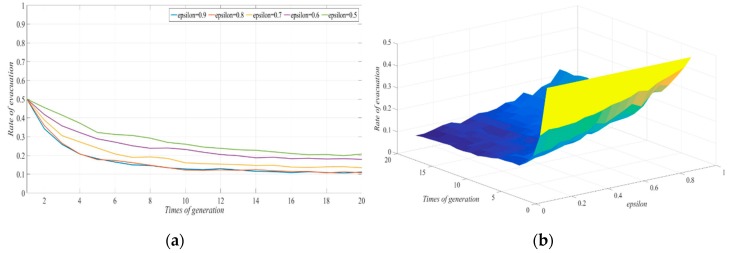
Effect of changes in ε on the evolution of evacuation systems: (**a**) Two-dimension; (**b**) three-dimension.

The other parameter values were fixed in order to investigate the impact of the degree coefficient of government participation resulting in the reduced consumption cost of the non-evacuation group to the original cost on the strategy selected by the evacuated group to evacuate under the condition of a typhoon warning and a government guarantee for both groups (one party evacuates while the other does not). We let ε be 0.9, 0.8, 0.7, 0.6, and 0.5. The evolutionary results of the strategy selection by the evacuated group in the social network are shown in Figure 4, with government involvement. With the continuous decrease in the rate of reducing the non-evacuation cost in the evacuation process to the original cost, the evacuation response rate in the social system gradually decreased. In addition, when ε was greater than 0.7, with the continuous exchange of information, the number of people in the evacuated group who chose not to evacuate increased, and the proportion of evacuated people was relatively small. As ε decreased to less than 0.7, the role played by government reinforcement gradually disappeared.

In the end, almost all of the evacuated groups in the social system chose to stay. This was because, in order to protect the safety of all personnel, the Government would take certain measures to ensure cost reduction for those who chose to evacuate and those who chose to stay. This is described as an anti-disaster campaign, which not only involves protecting people’s health and lives, but also mitigating the financial effect of a disaster (such as protect the fixed assets of the evacuees) as well as the delivery of materials and machinery to mitigate damage [35]. For the group that chose to stay, the role of government participation in providing a guarantee was reflected in the supply of emergency supplies and the maintenance of price stability in a special period, which would reduce the cost to a certain proportion under free-market conditions. That the results showed that the decision not to be evacuated by the evacuated group was more sensitive to cost. Even when the Government’s participation in the cost reduction of non-evacuation was not obvious, it would still promote the non-evacuation decision. When the guarantee adopted by the Government reached a certain level, the system would eventually evolve into a state of non-evacuation. Therefore, the Government’s guarantee of the distribution of emergency supplies under typhoon disaster conditions can play an important role in the decision not to evacuate. In order to avoid the “shadow evacuation” in typhoon evacuation, a certain degree of guarantee measures should be taken for relatively safe areas, which can free up more social resources to the evacuated group, thus ensuring the improvement in evacuation efficiency.

#### 4.2.4. The Degree Coefficient of Government Participation Reduced the Consumption Cost of the Non-Evacuation Group to the Original Cost (Considering the Non-Evacuation Group Only)

**Figure 5:**
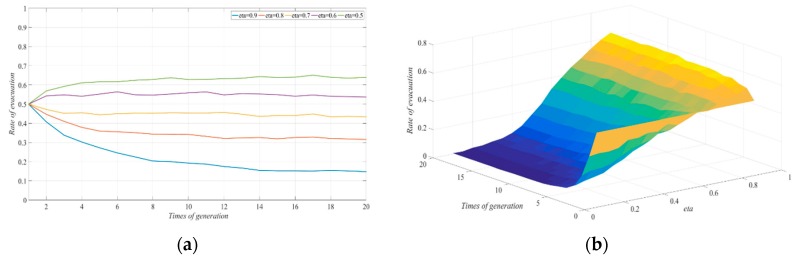
Effect of changes in η on the evolution of evacuation systems: (**a**) two-dimension; (**b**) three-dimension.

The other parameter values were again fixed in order to examine the impact of the degree coefficient on the evacuation strategy selection by the evacuated group under the typhoon warning conditions and with a full guarantee by the Government for the non-evacuation group (both parties do not evacuate) to reduce the consumption cost compared to the original cost. We let η be 0.9, 0.8, 0.7, 0.6, and 0.5; the evolutionary results of the strategy selected by the evacuated group in the social network are shown in Figure 5. With a continuous decrease in the rate of reducing the cost of non-evacuation in the evacuation process caused by government participation, the evacuation response rate in the social system gradually decreased. When η was 0.8–0.9, the proportion of evacuated groups was relatively high; when η was 0.5–0.7, the Government’s participation in reducing the cost of staying in the non-evacuation group had the effect of promoting the decision not to evacuate, thus reducing the evacuation rate in the social network. This was because in a relatively safe area, the Government was mainly committed to ensuring a reduction in the cost of non-evacuation groups. Therefore, when the intensity of the Government guarantee is relatively weak, the relatively high number of people who do not evacuate will result in a relatively high level of costs. Some people, therefore, will give up the non-evacuation strategy and choose to go to safe areas instead to reduce the cost of living in the special period. As the Government’s participation in guarantee increases, more people will choose not to evacuate.

It should be noted that although η and ε were similar, in that both reflected the reduction in non-evacuation costs due to government participation in providing a guarantee, the groups that chose to evacuate were more sensitive to the cost of non-evacuation under the condition (ε) of government participation in guaranteeing the interests of all groups. Under the condition (η) of guaranteeing the supply of materials for the non-evacuation group, if the Government did not adopt more effective guarantee measures, it would also cause the non-evacuation group to change its strategy after information exchange and choose to evacuate to a safe area. Therefore, the Government guarantee of the provision of emergency supplies under the conditions of typhoon disasters can play an important role in the decision not to evacuate. In order to avoid unnecessary panic caused by excessive estimates of disasters, it is necessary to avoid the situation of disasters causing panic buying of emergency supplies, which will increase prices, so as to guarantee social stability during a disaster.

#### 4.2.5. Impact of Reduction in Evacuees Leading to Cost-Reduction Rate of Non-Evacuation in the Evacuation Process

**Figure 6:**
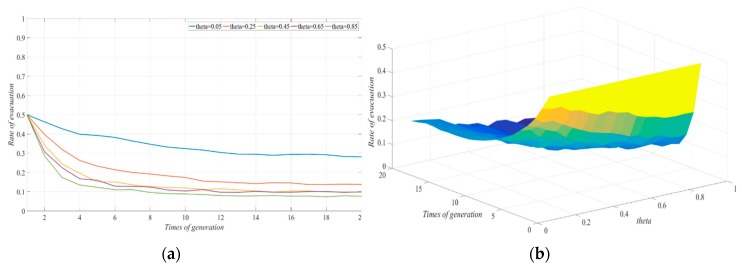
Effect of changes in θ on the evolution of evacuation systems: (**a**) two-dimension; (**b**) three-dimension.

The other parameters were fixed in order to explore the impact on the selection of the non-evacuation strategy caused by the reduction in evacuees leading to differences in the cost-reduction rate of non-evacuation in the evacuation process under the typhoon warning condition. We let θ be 0.05, 0.25, 0.45, 0.65 and 0.85 and observed the impact of changes in θ on the evolution of the system. As can be seen from Figure 6, when the number of evacuees increased, the cost-reduction rate of non-evacuation increased, and the evacuation response rate in the social system gradually decreased. When θ was 0.05, some in the evacuation group gradually chose the non-evacuation strategy after the information exchange, and the evacuation response rate in the social system was reduced; when θ was 0.25–0.85, eventually, all in the social system would choose the non-evacuation strategy.

Moreover, savings in the non-evacuation cost (θ) of social resources given by the people who chose the strategy to evacuate had a more significant effect on the evacuation decision than the cost savings afforded by policy participation (ε). Similarly, when the savings in the emergency supplies cost caused by the increase in the evacuation groups reached a certain level, its stimulating effect on the evacuation decision gradually disappeared, and eventually, all the evacuated groups in the social system would choose to stay. This was because in the case of limited social resources, when the evacuated group chose the evacuation strategy, it would give up certain emergency support resources and save certain costs for the individual who chose the non-evacuation strategy. As long as the savings in the cost reached a certain level, then in the case that the probability of typhoon disaster was relatively low, the individual was more inclined to stay than evacuate. After continuous information exchange, the groups in the social system tended to choose not to evacuate.

## 5. Conclusions

As the risk of global extreme weather events continues to increase, effectively improving emergency evacuation efficiency and ensuring the safety of personal property has become a constant concern for governments and the public. A full understanding of the internal patterns of public evacuation decisions can help the Government scientifically develop an emergency evacuation plan to improve the public’s compliance with evacuation orders. This study mainly analyzed the decision-making mechanism of the evacuated groups, the impact of individual interests, other people’s decision-making, and the Government’s evacuation policy on evacuation decision-making. We then simulated and analyzed the impact of different factors on the decision diffusion of an emergency evacuation in the whole social system by constructing an evolutionary game model of complex networks.

The predictability of typhoon disaster provides the opportunity for the adoption of prevention activities especially requiring efficient evacuation of residents from hazardous areas within a specific time window. No matter evacuate or stay at home, effective prevention activities can leading to the progressive reduction of the effects of a disaster can cause to humans, buildings and the environment [47]. This process requires government intervention to provide appropriate guidance and make certain guarantees. The results of this study indicated that the Government’s participation in reducing the evacuation cost can promote evacuation strategy by the evacuated groups. This will be reflected in the Government’s effective and reasonable evacuation strategy. Participation in evacuation guidance can also play an important role in reducing evacuation costs; Government participation in reducing the non-evacuation cost can promote a non-evacuation strategy by the evacuated groups. This may be reflected in the Government’s planning more effectively, mobilizing emergency materials in a short period of time, and improving emergency supplies to reduce non-evacuation costs. At the same time, the Government can protect some of the fixed assets of the evacuees.

The above conclusions provide support for the Government’s policy recommendations for promoting emergency evacuation decisions for typhoons. When the risk of a typhoon is high, it is necessary to actively participate in evacuation work, take effective measures to help ease traffic, and help reduce evacuation costs, including support for the evacuation of vulnerable groups, who often incur high evacuation costs. On the one hand, providing evacuation vehicles can effectively alleviate the lack of road network capacity and help groups without cars evacuate; on the other hand, it is necessary to have emergency facilities and emergency supplies at refuge stations to help reduce the evacuation cost of the evacuated groups. Second, while helping the evacuation operations proceed in an orderly manner, the Government also needs to pay attention to the groups who are in relatively safe areas and do not need to evacuate, promptly mobilize resources for emergency guarantees, ensure the supply of emergency materials, and release disaster-related information and clear early evacuation warnings promptly to minimize the risk of inappropriate evacuation and resulting road blockages. Third, the Government should continuously improve the application level of traffic simulation technology and Geographic Information System (GIS) technology, accurately estimate the evacuation path and typhoon risk, make emergency evacuation plans, improve risk-response capabilities, and formulate policies such as post-disaster reconstruction and catastrophe insurance to protect the fixed assets of the evacuees and relieve the worries of the evacuated groups. It should be noted, though almost all of the coastal cities in China have typhoon evacuation plans. The most important thing is not only making the scientific plan, but also making all the participants know how to react during or before different grades of typhoon disaster.

There were some limitations to this study. For instance, we drew relevant conclusions based on a simulation analysis. The setting of relevant simulation data came from literature or expert experience. However, studies of actual data in this simulation model are needed to conduct prediction analysis. Such data can improve the accuracy of the findings in this study. In the future, other research methods such as survey investigation should be used to obtain the data of evacuation demand under different conditions. In addition, structural equation models should be used to explore the influence of factors on the evolution of evacuation systems in order to improve the accuracy of parameter values put into the simulation.

## Figures and Tables

**Table 1:** Payoff Matrix of the Model.

Group		Evacuees-G2	
		Evacuate	Stay
Evacuees-G1	Evacuate	$U_{11}$; $V_{11}$	$U_{12}$; $V_{12}$
	Stay	$U_{21}$; $V_{21}$	$U_{22}$; $V_{22}$

**Table 2:** Analysis of Equilibrium Points.

*(x, y)*	*Det J*	*Tr J*
(0,0)	$A_{2}B_{2}$	$A_{2}+B_{2}$
(0,1)	$-(A_{1}+A_{2})B_{2}$	$A_{1}+A_{2}-B_{2}$
(1,0)	$-A_{2}(B_{1}+B_{2})$	$-A_{2}+(B_{1}+B_{2})$
(1,1)	$\left(A_{1}+A_{2})(B_{1}+B_{2}\right)$	$\left(A_{1}+A_{2})+(B_{1}+B_{2}\right)$
(x *, y *)	$-xy(1-x)(1-y)A_{2}B_{2}$	0

Note: * represents the saddle-points of system.

**Table 3:** Values of Fixed Parameters in the Payment Matrix.

Network Structure	Network Size	Number of Iterations	α	*P*_1_	*C*_1_	${C_{1}}^{\prime }$	*D*_1_	*E*_1_
				10	8	1	4	2
Small-world	200 nodes	20	0.5	***P*_2_**	***C*_2_**	${C_{2}}^{\prime }$	***D*_2_**	***E*_2_**
				**10**	**8**	**1**	**4**	**2**
				β	θ	δ	ε	η
				0.05	0.05	0.1	0.9	0.9
				0.25	0.25	0.2	0.8	0.8
				0.45	0.45	0.3	0.7	0.7
				0.65	0.65	0.4	0.6	0.6
				0.85	0.85	0.5	0.5	0.5
